# Feasibility and Acceptability of a Real-Time Adherence Device among HIV-Positive IDU Patients in China

**DOI:** 10.1155/2013/957862

**Published:** 2013-07-16

**Authors:** Mary Bachman DeSilva, Allen L. Gifford, Xu Keyi, Zhong Li, Cheng Feng, Mohamad Brooks, Mark Harrold, Hu Yueying, Christopher J. Gill, Xie Wubin, Taryn Vian, Jessica Haberer, David Bangsberg, Lora Sabin

**Affiliations:** ^1^Center for Global Health and Development, Boston University School of Public Health, 801 Massachusetts Avenue, Crosstown, 3rd Floor, Boston, MA 02118, USA; ^2^Department of Health Policy and Management, Boston University School of Public Health, Talbot Building, T348W, Boston, MA 02118, USA; ^3^Ditan Hospital, 13 Ditan Park, Andingmen Outer Street, Beijing 100011, China; ^4^FHI 360, Asia-Pacific Regional Office, 19th Floor, Tower 3, Sindhorn Building, 130-132 Wireless Road, Kwaeng Lumpini, Khet Phathumwan, Bangkok 10330, Thailand; ^5^Global Health Strategies, Manila Room, 17th Floor, Ping an International Financial Plaza, Tower B, No. 1-3 Xinyuan South Road, Chaoyang District, Beijing 100022, China; ^6^Guangxi CDC ART Clinic, 18 Jinzhou Road, Nanning, Guangxi 530028, China; ^7^Center for Global Health, Massachusetts General Hospital, 100 Cambridge Street, 15th Floor, Boston, MA 02114, USA

## Abstract

We collected data on feasibility and acceptability of a real-time web-linked adherence monitoring container among HIV-positive injection drug users (IDU) in China. “Wisepill” uses wireless technology to track on-time medication dosing. Ten patients on antiretroviral therapy (ART) at the Guangxi CDC HIV clinic in Nanning, China, used Wisepill for one ART medication for one month. We monitored device use and adherence and explored acceptability of the device among patients. Mean adherence was 89.2% (SD 10.6%). Half of the subjects reported a positive overall experience with Wisepill. Seven said that it was inconvenient, supported by comments that it was large and conspicuous. Five worried about disclosure of HIV status due to the device; no disclosures were reported. Twelve signal lapses occurred (5.4% of prescribed doses), of which one was due to technical reasons, nine to behavioral reasons (both intentional and unintentional), and two to unclear reasons. Although the technical components must be monitored carefully, and acceptability to patients presents challenges which warrant further exploration, the Wisepill device has potential for adherence interventions that deliver rapid adherence-support behavioral feedback directly to patients, including IDU. The use of wireless technology appears uniquely promising for providing time-sensitive communication on patient behavior that can be harnessed to maximize the benefits of HIV treatment.

## 1. Introduction

Interventions to improve adherence to antiretroviral therapy (ART) are urgently needed among HIV-positive patients, particularly in developing countries, where most of the world's HIV-infected population lives. China has experienced a major HIV/AIDS epidemic since the 1990s, with an estimated 780,000 persons living with HIV in 2011 [1]. Free ART is available nationwide and over 126,000 Chinese patients are now on treatment. However, few ART adherence studies have been conducted in China. As it is elsewhere in the world, ART adherence is suboptimal in China in large part because HIV/AIDS is highly stigmatized [2, 3].

Recently, wireless technology has emerged as a potential tool for monitoring medication adherence in real time [4–8]. This is noteworthy given the recognized association between late dose-timing and loss of viral suppression [9]. One promising tool, the Wisepill personal medication container (Wisepill Technologies, Cape Town, South Africa), detects the exact date and time whenever the patient opens it to access medication. It then transmits a real-time signal by general packet radio service (GPRS) to a central server where the data are recorded securely and accessible to appropriate clinicians or researchers. Wisepill and other similar devices are currently being pilot tested in various countries for different uses, such as infection control and oral hygiene [10, 11]. Although these devices have the potential to support adherence monitoring and counseling, few studies have assessed feasibility and acceptability among ART patients, particularly in developing countries [12, 13].

In a previous intervention study (Adherence for Life (AFL)), which tested the use of electronic drug monitoring (EDM) data as an information and counseling tool among primarily injection drug using (IDU) ART patients in China's Yunnan province, we found that monthly EDM-informed counseling significantly improved mean ART adherence and CD4 counts [14]. These findings indicate that use of electronic data-collecting pill containers is feasible in China and offer proof of concept that providing Chinese patients and providers with adherence data can positively impact patients' adherence.

To lay the groundwork for a larger intervention study in China using wireless technology to monitor adherence and intervene in real time among injection drug users (IDU), we conducted an in-depth feasibility and acceptability study on the use of the device in a small cohort of IDU patients. This approach allowed us to glean important information about the potential for use of the device given the sociocultural circumstances of provision of ART in China, including the following: HIV is highly stigmatized; ART is relatively new, and policies on provision have changed in recent years; use of cellphone technology, while ubiquitous and familiar, is in rapid transition; and the clinician-patient relationship, particularly when the patient is an IDU, is extremely hierarchical. 

## 2. Materials and Methods

### 2.1. Study Site and Population

The study was led by Boston University's Center for Global Health and Development (CGHD), with enrollment at the Guangxi Centers for Disease Control (CDC) antiretroviral therapy (ART) clinic in Nanning, China, a large clinic that currently treats over 1,400 patients, including 1,040 adults, 370 children, and a high proportion of IDU among the adult patients. We provided ten current or former IDU patients who were being treated with ART at the clinic with a Wisepill device for one or more of their ART medications. We then monitored their use of the device for one month without the subjects receiving any information about their adherence data. This allowed us to collect pilot data on (1) acceptability of use of the Wisepill device among a Chinese patient population and (2) feasibility of monitoring patient adherence using the device in China. We limited the sample size to ten subjects due to budget constraints and to permit a rapid assessment prior to initiating the larger study, but often formative feasibility studies of this type have used similar sample size [12, 15].

### 2.2. Data

The study involved the following sources of patient data: (1) a baseline sociodemographic and health history questionnaire; (2) a brief self-report of adherence and use of Wisepill from subjects after 1 month of use; (3) continuous adherence data from the Wisepill device; (4) continuous data on reasons for signal lapses; and (5) CD4 and VL test results from patients' medical charts.

### 2.3. Sociodemographic and Wisepill Experience Data

The baseline and monthly interviews were administered in Mandarin Chinese by trained clinic staff. In addition to sociodemographic information and health history, the baseline instrument also covered route and duration of HIV infection, history of depression, and alcohol/drug use. Besides self-reported adherence, the monthly form included quantitative and open-ended questions covering acceptability and usability of the Wisepill device (ease/convenience of use, difficulties using the device, device storage, and potential stigma/loss of confidentiality), as well as perceptions of a possible intervention that makes use of Wisepill.

### 2.4. Wisepill Medication Dispenser and Adherence Monitoring Device

**Figure 1:**
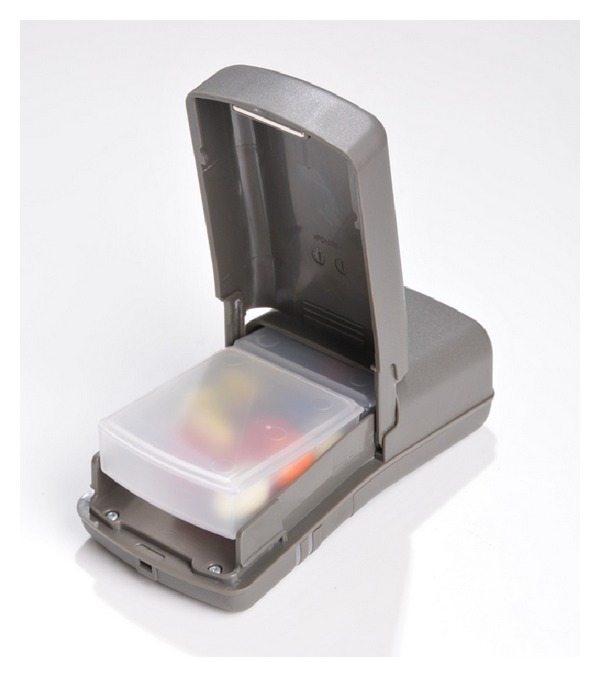
The Wisepill device.

The Wisepill device measures 30 × 60 × 130 mm and holds up to 60 small pills in two inner compartments (see Figure 1). It is powered by one rechargeable 3.7 volt 1100 mAh lithium polymer battery and contains a Subscriber Identity Module (SIM) card. The device creates a date and time stamp each time it is opened and transfers this information by general packet radio service (GPRS) to a central server in South Africa. The data are then available to research, clinic, and program personnel via a secure, internet-based interface.

### 2.5. Adherence Data and Measures

Data on Wisepill openings were transmitted automatically and continuously over the month, as described previously. Investigators downloaded the data from a password-protected account on the Wisepill website. From these data, we calculated mean adherence over the one-month period using the following formula: (number of doses taken ± one hour of dose time)/(number of prescribed doses); the approach used in the AFL study based on the adherence measure that was most significantly associated with viral load [16]. We also analyzed the adherence data self-reported by subjects in the monthly form using a visual analog scale (VAS) that indicates the proportion of total doses patient took and compared this with the adherence data generated by the Wisepill device.

### 2.6. Signal Lapses

We investigated all Wisepill signal lapses by a phone call to the subject within 2-3 days to determine whether a lapse was due to technical failure (battery failure, forwarder malfunction) or had a behavioral cause (missed dose, intentional nonuse). This information was recorded in a “signal lapse” report that we created for each subject over the month of data collection. We then calculated separately the proportion of technical issues and behavioral reasons among all expected Wisepill signal lapses.

### 2.7. Clinical Data

CD4 and viral load test results were collected from subjects' medical charts as background information on subjects; no additional blood draws were required for the study. 

### 2.8. Data Analysis

For all quantitative data, we calculated descriptive statistics (means, ranges, standard deviations for continuous variables, and frequencies for categorical variables). Qualitative data from the open-ended questions were analyzed using a thematic approach. All quantitative analyses were conducted using SAS 9.1 (The SAS Institute, Cary, NC, USA). 

The study was approved by the Institutional Review Boards at Boston University Medical Center and the Guangxi Provincial Center for Disease Control, Nanning, China. All subjects provided written informed consent prior to enrollment. Because it was not a clinical trial, registration with http://ClinicalTrials.gov/ was not required.

## 3. Results

### 3.1. Participants

The ten subjects were current and former injection drug users, and most (7) reported that they had been infected via shared needles. The mean age of the sample was 32.7 years (SD = 5.3); seven of the ten were men, and six were married. On average, the subjects had been on ART 40.9 months (SD = 29.4) and had a mean baseline CD4 count of 383 cells/*μ*L (SD = 170 cells/*μ*L). Seven had a history of depression, and two were alcoholic. Four reported having hepatitis C, and two reported having had syphilis. Nine of the ten had an educational level of middle school or lower. Most subjects had a low monthly income, typical of IDU in China. While one did not know and one reported a monthly income of 1001–5000 Yuan (approximately $ 159–794), eight had a monthly income of 1000 Yuan (approximately $ 156) or less. 

### 3.2. Wisepill Data Transmission and Adherence Lapses

The total prescribed (expected) number of device openings was 614 for the month of study. Twelve lapses occurred over the month (33 total doses); thus 33/614 or 5.4% of prescribed doses were not recorded in real time. The mean duration of real-time lapses was 2.75 doses (range 1 dose–21 doses). 

Of the lapses, one was due to technical reasons, nine to behavioral reasons, and two to unclear reason. The technical problem was a lack of airtime on the SIM card, but this resulted in 21 consecutive openings missed because the subject could not be contacted. After the SIM card was reactivated, these initially missed doses were eventually received by the server, so they could be included in the subject's adherence calculation. If these 21 openings not recorded in real time are included, the proportion of openings not measured by the device was 2.0% (12/614). Of the nine behavioral lapses, seven were due to a subject forgetting to take a dose, and one was due to a subject forgetting to close the device after use. The final behavioral lapse was due to a patient purposely not using the box (reportedly taking a dose out early and actually taking the medicine later at work). 

### 3.3. Adherence Levels

Using Wisepill data, adherence was 97.2% (SD = 3.5%) of prescribed doses taken and 89.2% (SD = 10.6%) using a measure that incorporates dose timing (detailed previously). Using a visual analogue scale at the monthly visit, self-reported adherence was 98.5% (SD = 3.2%). 

### 3.4. Acceptability of Wisepill Device

In quantitative questions, half of the subjects reported a positive or very positive overall experience with Wisepill; the other five reported a “somewhat negative” overall experience. Seven were willing or very willing to participate in a larger intervention study. Eight found the device very easy to use. However, seven said that it was inconvenient or very inconvenient. Five were somewhat or very worried about disclosure of their HIV status due to the device; no disclosures were reported. 

#### 3.4.1. Ease and Convenience of Use

In the open-ended questions exploring acceptability and usability of the Wisepill device (ease/convenience of use, difficulties using the device, device storage, and potential stigma/loss of confidentiality), six patients reported a positive feeling about the device, of whom four said knowing that someone was monitoring their adherence helped them take their medications more regularly. As two subjects explained:

 Knowing that someone is monitoring my medication spurs me to take my medication better. The pill box is just a normal drug container; there is nothing good or bad about it.
 I like the pill box, because first of all, there is no special label on the pillbox (comparing with the medicine bottle, on the instruction label there is information about HIV medicine), so no one will know what medicine I am taking; second, it records the time of medicine taking, which helps me take doses better.

Three reported a negative experience. One said the device was inconvenient to carry and therefore a burden, and one did not like the feeling of being watched (this one had both positive and negative feelings as he/she also said using the device was a helpful reminder).

#### 3.4.2. Difficulties of Use Including Potential Stigma and Loss of Confidentiality

When asked about difficulties encountered using the Wisepill device, one subject reported having no problem with the device at all, while another had no major problem because he/she “always carried it in a bag.” However, eight indicated that the device was inconvenient or felt uncomfortable using it. Many found it too big; one patient thought it should be wider. Additional feedback included the following: two subjects did not like that it could only hold one drug; two worried that drugs would be damaged while carrying the device; two were concerned about others seeing it. These were typical statements:

 It is too big to carry. I have a feeling of unease using the Wisepill in front of other people. I never take the pillbox outside.
 The pillbox is too big. I have a big concern that using this pill box could disclose my HIV status.

#### 3.4.3. Device Management and Storage Strategies

Subjects reported a variety of ways to manage their use of the device. One compensated for the inconvenience by always taking doses at home, another changed his/her dose time to avoid carrying the device to work, and one told friends that the drugs were a hepatitis medicine instead of HIV-related medications. Seven of the patients reported keeping their device at home exclusively; six seemed to keep it hidden, whether at home or outside. Patients were also asked what they did with the device when they traveled. Half reported never taking the device away from home; the other half reported having found ways to travel with the device. One of the former elaborated that

 I have no job in the past one month. I always take my medicine at home. Even when I go to parties or meeting friends, I did not bring the pill box with me. That's why, sometimes when I returned home late, I also took my medications late.

#### 3.4.4. Reminder Messages and Willingness to Participate in a Larger Study

When asked specifically about text messages, only four patients thought reminder messages would be helpful. Of the six who did not, one was a truck driver and could not read messages while driving, one did not read text messages at work, and one thought that text message reminders are not much more useful than an alarm.

When asked about possibly participating in a larger study using Wisepill with text message reminders, six said they would be willing to participate, of whom four said it would help them take their ART medications on time. As two explained:

 Yes, the reminder message could help. I usually read short messages. I hope the message could be as simple as possible, like a symbol would be good.
 Yes, the reminder message could help. The SMS could just be “It's time to take your medication, do not forget”. I do not worry about other people (knowing) my health status through this message.

Three subjects were not willing to participate in a subsequent larger study. All three did not think text messages would work; two said the box was too inconvenient. One was concerned about possible disclosure of status via messages, and one did not use text messages.

## 4. Discussion

This study has demonstrated that use of a real-time, SMS-enabled web-linked ART adherence monitoring system is technically feasible in an urban Chinese clinical setting among predominantly IDU HIV patients. Only a few minor technical difficulties were encountered and easily addressed; the issue of acceptability is more complex.

The technical lapses due to inadequate time on the SIM card bear some discussion. In terms of adherence measurement, these types of lapses do not represent a major concern; as long as there is adequate battery power in the device, the openings are recorded but are just not transmitted to the server until the airtime on the SIM card is replenished. In other words, the transfer of information is delayed, but the data needed for characterizing adherence rates are not affected. Because the system did not work perfectly for capture of data in real time, however, this presumably would have an impact on its effectiveness as a tool for promoting adherence, including triggered SMS reminders. Moreover, if an intervention was designed to send an SMS message when the server does not receive a signal within a set time window, then the server would send an SMS reminder to the participant regardless of the reason for the lapse. In such an intervention context, the subject with the lapse of 21 doses in the present study would have received 21 reminder messages even though she/he actually used the device correctly and took all of the doses on time. Readers in the USA should also note that in most other countries including China, a mobile phone that has run out of SIM card airtime can still receive text messages. The main risk here is that patients might become annoyed. In short, the logistical requirements of the device are real (airtime, out of range issues, and battery power) and can cause some problems. A technical lapse due to the airtime issue does not preclude us from measuring and understanding adherence behavior, but to take full advantage of the technical capacity of the device, researchers and clinicians must pay close attention to these logistical requirements.

The study raises greater concerns about the acceptability of the device to patients. Several subjects complained about the size and inconvenience of the device. To overcome these concerns, consideration should be given to the design and appearance of the device. Some ideas might include devising a carrying case or bag to make the device less conspicuous or creating a cell phone case that would hold both the phone and the monitoring device. A smaller less conspicuous box, perhaps retaining the removable pill containers for easy refilling that Wisepill currently offers, might also be considered. In addition, although no disclosures were reported during the pilot, subjects did raise concerns about disclosure and stigma due to the device in both closed and open-ended questions. This is a potential problem with the device which deserves further attention [8]. That said, the extent to which even our small sample of patients devised a variety of ways of using the device is striking. Input from patients with experience with the device will be critical to thinking about how to make it easier and potentially less stigmatizing to use in daily life. 

A limitation of the study is the small sample of ten individuals who used the device, with no accompanying intervention that might foster more positive feelings about the device. The full wireless capabilities of the device were not tested and could not be appreciated. It is possible that subjects' attitudes might change after having the experience of receiving SMS reminders, particularly if they could see their adherence increase or their health improve as a result. 

The technical findings from the study are more persuasive, but the qualitative aspects are by definition limited and subjective. Therefore, we revised aspects of the subsequent larger randomized controlled trial currently underway in the same clinic, including our data collection instruments, to allow more extensive collection of qualitative data. This will allow us to obtain more conclusive data from the larger study, in which intervention subjects receive a tailored SMS reminder message if they are more than 30 minutes late taking a dose. Those who are suboptimal adherers (<95%) also review a printout of their adherence behavior over the previous month in counseling sessions. The design of this larger study will permit us to collect quantitative and qualitative data on acceptability over time for both intervention and control groups and thereby gain a deeper understanding of the evolution of acceptability of the device over time in this Chinese patient population.

## 5. Conclusions

Although the technical logistical requirements must be monitored carefully, and acceptability to patients is not perfect, the Wisepill device shows potential for adherence interventions that deliver rapid adherence-support behavioral feedback directly to patients, including IDU, as well as in clinical settings. The fact is that each current adherence monitoring device or technology has its advantages and disadvantages, as well as a certain measure of intrusion for patients. Wisepill involves a high degree of intrusion but also a high degree of accuracy as well as the unique benefit of real-time monitoring which allows for real-time interventions to improve adherence before the substantial negative impact of poor adherence can accumulate to cause substantial harm. Other adherence measures (self-report, pill count, pharmacy refill, e.g.) may involve a lesser burden for patients, but they are less accurate and do not permit real-time interventions. Electronic drug monitoring reviewed at the time of clinic visit (such as the Medication Event Monitoring System (MEMS)) imposes a similar degree of intrusion on patients compared to Wisepill, but again, there is no opportunity to intervene in real time. Thus, while the Wisepill delivery system is not perfect, we believe that it holds substantial advantages over other currently available adherence monitoring options. Just as so many recent technological advances offer the possibility of client-centered approaches, the use of wireless technology appears uniquely promising for providing time-sensitive communication on patient behavior that can be harnessed to maximize the benefits of HIV treatment.
